# Criterion and incremental validity of the emotion regulation questionnaire

**DOI:** 10.3389/fpsyg.2015.00247

**Published:** 2015-03-11

**Authors:** Christos A. Ioannidis, A. B. Siegling

**Affiliations:** ^1^Division of Psychology and Language Sciences, University College LondonLondon, UK; ^2^London Psychometric Laboratory, Research Department of Clinical, Educational and Health Psychology, University College LondonLondon, United Kingdom

**Keywords:** emotion regulation, emotion regulation strategies, emotion regulation questionnaire, incremental validity, criterion validity

## Abstract

Although research on emotion regulation (ER) is developing, little attention has been paid to the predictive power of ER strategies beyond established constructs. The present study examined the incremental validity of the Emotion Regulation Questionnaire (ERQ; Gross and John, 2003), which measures cognitive reappraisal and expressive suppression, over and above the Big Five personality factors. It also extended the evidence for the measure's criterion validity to yet unexamined criteria. A university student sample (*N* = 203) completed the ERQ, a measure of the Big Five, and relevant cognitive and emotion-laden criteria. Cognitive reappraisal predicted positive affect beyond personality, as well as experiential flexibility and constructive self-assertion beyond personality and affect. Expressive suppression explained incremental variance in negative affect beyond personality and in experiential flexibility beyond personality and general affect. No incremental effects were found for worry, social anxiety, rumination, reflection, and preventing negative emotions. Implications for the construct validity and utility of the ERQ are discussed.

## Introduction

Emotion regulation (ER) refers to “processes by which we influence which emotions we have, when we have them, and how we experience and express them” (Gross, 2002, p. 282). ER strategies rely on the particularity of the situation and do not assume an intrinsically positive or negative character (Gross, 2002). It is, however, possible to differentiate between healthy and unhealthy strategies on the basis of some yielding more beneficial outcomes than others in the long run (John and Gross, 2004). By extension, individuals can develop adaptive or maladaptive emotion regulatory styles through their tendency of using some strategies more than others (Phillips and Power, 2007).

Research on ER during the last two decades has primarily focused on its role in health and psychopathology. Emotion dysregulation appears related to maladaptive functioning (Aldao et al., 2010), numerous psychological disorders (e.g., Gross, 1999; Cisler et al., 2010), and was even found to have implications for physical health (Pennebaker, 1990; Siegeman, 1993). Yet, the explanatory power of ER measures and, in particular, measures of specific ER strategies beyond relevant established constructs has remained largely unexplored. The majority of ER measures either assess generalized, strategy-independent ER (Gratz and Roemer, 2004), or are limited to examining regulatory strategies based on isolated elements of emotional arousal, such as cognition (e.g., Garnefski et al., 2001). The present investigation examines the criterion and incremental validity of the Emotion Regulation Questionnaire (ERQ; Gross and John, 2003), which measures two broad ER strategies that affect different aspects of emotion.

### The ERQ

The ERQ was developed by Gross and John (2003) for the purpose of measuring two ER strategies, one considered beneficial and the other harmful. According to the authors, regulatory strategies can be broadly categorized as antecedent-focused and response-focused, depending on the point at which the individual intervenes in emotional processing. Cognitive reappraisal (CR) is an adaptive, antecedent-focused strategy, affecting the early cognitive stages of emotional activity, whereby the initial interpretation of a given situation is re-evaluated. In contrast, expressive suppression (ES) is a maladaptive, response-focused plan of action implemented after an emotional response has fully developed; it is conceptualized as inhibiting the behavioral expression of the emotion. The ERQ concentrates on these two broad strategies, measuring the tendency of individuals to use CR and ES as strategies of dealing with emotional arousal.

Since its development, the ERQ has been widely used in studies of ER and has presented good psychometric properties. In particular, the measure has demonstrated adequate to good internal consistency and temporal stability (Gross and John, 2003; Sala et al., 2012; Batistoni et al., 2013), as well as sound convergent and discriminant validity (Gross and John, 2003; Balzarotti et al., 2010; Cabello et al., 2012). The two-factor structure (CR and ES) proposed by Gross and John has been replicated by most investigations (Balzarotti et al., 2010; Cabello et al., 2012; Uphill et al., 2012), while encouraging results have been reported regarding measurement invariance and factor distinction (Moore et al., 2008; Melka et al., 2011; Sala et al., 2012).

### Criterion validity of the ERQ

The criterion validity of the ERQ has been intensely studied, revealing numerous associations with constructs related to adaptive and non-adaptive functioning. Initial studies by Gross and John (2003) showed CR to be related to greater positive affect (*r* = 0.42), mood repair (*r* = 0.36), and life satisfaction (*r* = 0.30), as well as to reduced negative affect (*r* = −0.51) and depression (*r* = −0.23 to −0.29). In contrast, ES was negatively associated with positive affect (*r* = −0.33), mood repair (*r* = −26), and life satisfaction (*r* = −0.34), while correlating positively with negative affect (*r* = 0.39), depression (*r* = 0.23 to 0.27), and inauthenticity (*r* = 0.47).

The findings involving affect have been a matter of controversy, since the same relationships are not consistently found. Contradicting evidence from later investigations (Balzarotti et al., 2010; Cabello et al., 2012; Batistoni et al., 2013) mainly concerned associations of ER strategies with negative affect, but these studies were conducted on culturally different samples (Italian and Spanish) than the initial US study. Cabello et al. (2012) posited that the difference between American and Italian or Spanish data might result from the more collectivist character of southern European cultures influencing the relationship between ER and negative affect. However, following the most recent country classification in terms of Hofstede's cultural dimension of individualism vs. collectivism (Hofstede et al., 2010), Spain can be considered as culturally neutral, while Italy is found closer to the individualist end of the spectrum. Meanwhile, some studies (Kashdan et al., 2006; Batistoni et al., 2013) have also reported non-significant relationships of positive affect both with CR and ES showing that inconsistent results are not limited to negative affect.

Apart from irregular findings for affect, most of the above results have been largely replicated (Kashdan et al., 2006; Wiltink et al., 2011; Cabello et al., 2012; Batistoni et al., 2013). Moreover, generalized anxiety and rumination (Gross and John, 2003; Wiltink et al., 2011) were found to correlate positively with ES (*r* = 0.08 to 0.19) and negatively with CR (*r* = −0.10 to −0.29), while positive relations with others (Gross and John, 2003; Cabello et al., 2012; CR = 0.17 to 0.23; ES = −0.34 to −0.46), social support (Cabello et al., 2012; CR = 0.12; ES = −0.28), self-esteem (Gross and John, 2003; CR = 0.30; ES = −0.39) and curiosity (Kashdan et al., 2006; CR = 0.20; ES = −0.37) were all found to correlate negatively with ES and positively with CR. Finally, social anxiety was positively associated with ES (*r* = 0.28; Kashdan et al., 2006).

### Incremental validity of the ERQ

Gross and John (2003) reported that all Big Five personality factors apart from Neuroticism correlate positively with CR and negatively with ES, and that CR correlates negatively with Neuroticism. Both of these results have been replicated (Gross and John, 2003; Balzarotti et al., 2010; Cabello et al., 2012; Gresham and Gullone, 2012; Páez et al., 2013). Results for Neuroticism and ES have been inconsistent; some investigations reported a non-significant relationship between ES and Neuroticism (Gross and John, 2003; Páez et al., 2013), while others found a positive association (Gresham and Gullone, 2012), and yet a negative association (Balzarotti et al., 2010; Cabello et al., 2012).

Evidence on the relation of the ERQ subscales with the Big Five personality factors suggests that ER strategies are associated, but not redundant with the Big Five (Gross and John, 2003; Balzarotti et al., 2010; Cabello et al., 2012; Gresham and Gullone, 2012; Páez et al., 2013). Correlations are consistently between small and moderate in size, suggesting that a still sizable portion of their variance is unaccounted for by higher-order personality factors. This limited convergence has been attributed to the Big Five capturing, by virtue of their conceptualization, a wider range of psychological phenomena than specific ER strategies (Gross and John, 2003). Hence, despite some overlap, personality and ER may play separate roles in psychopathology, and use of ER strategies should account for variance beyond higher-order personality factors in a range of construct-relevant criteria.

To the authors' knowledge, only two studies have examined the incremental validity of the ERQ. In an investigation by Páez et al. (2013), ES explained incremental variance in happiness and psychological well-being, controlling for the Big Five, alexithymia, daily mood, and humor style. In contrast, CR did not account for incremental effects in any of the criteria. The second study (Bardeen and Fergus, 2014) examined the incremental validity of generalized emotion dysregulation, measured with the Difficulties in Emotion Regulation Scale (Gratz and Roemer, 2004), in predicting health anxiety beyond use of specific ER strategies, assessed with the ERQ, and negative affect. Both ERQ subscales did not explain unique variance in the presence of generalized emotion dysregulation, and only ES made an incremental contribution when six generalized emotion dysregulation subscales were added separately. Nonetheless, the authors suggested that, since the Difficulties in Emotion Regulation Scale and ERQ measure different dimensions of ER, the suitability of an ER measure may depend on the criterion under investigation.

As ER research is still at a relatively early stage, further psychometric evidence is needed before strong conclusions about the validity of the construct and its measures can be drawn. The integration of adaptive ER strategies in clinical practice (e.g., CR in cognitive behavioral therapy; Clark, 1999) illustrates the practical need for continued research surrounding the measurement of ER strategies. Research into the incremental validity of ER strategy measures beyond cognate constructs implicated in psychopathology (e.g., emotional intelligence, trait affect) as well as more general trait constructs is critical in order to demonstrate their usefulness and justify their consideration in research applied contexts. Simultaneously, the limited evidence base for the criterion and predictive validity of ER strategies needs to be replicated and expanded.

### The present study

This investigation aimed to examine the associations of the ERQ subscales with emotion-laden and cognitive criteria as well as their incremental validity in predicting these criteria over the Big Five personality factors. The criteria included construct-relevant variables that have not been previously investigated as criteria of the ERQ and variables requiring replication for their documented relationship with the ERQ to be established. As unexplored constructs, reflection and experiential flexibility were utilized. Evidence regarding the relationship between curiosity and the ERQ subscales (Kashdan et al., 2006) indicates that the inquisitive introspective activity characterizing reflection should be positively predicted by CR and negatively by ES. Furthermore, Gross and John (1998) described the construct of inauthenticity as “perceived discrepancies between the inner experience and the outer expression of emotion or attempts at masking the expression of one's inner feelings for self-presentational purposes” (p. 175), a definition that is highly similar to that of experiential avoidance (Hayes et al., 2004). Judging from their theoretical resemblance, experiential flexibility (vs. avoidance) should show the opposite relationship than inauthenticity, correlating negatively with ES and non-significantly with CR.

This study also investigated the relation of ER strategies to mindful coping, or coping skills rooted in mindfulness qualities, which was selected in light of the rising empirical interest in the mindfulness construct and its theoretical relations to ER (Kumar, 2002). Since CR and ES have been positively and negatively related to active attempts at repairing negative emotional experiences, respectively (Gross and John, 2003; Cabello et al., 2012), the skill of preventing negative emotions should be negatively predicted by ES and positively predicted by CR. In addition, and as discussed, research supports the positive association of CR with self-esteem, positive relations with others, and social support, and the reversal of these relations for ES (Gross and John, 2003; Cabello et al., 2012). Therefore, constructive self-assertion, or maintaining successful relationships while also supporting one's self-regard, should demonstrate a similar pattern of associations, thus correlating positively with CR and negatively with ES.

With regard to researched criteria, the study attempted to replicate the observed relationships of the ERQ subscales with personality, affect, rumination, and anxiety. Two subtypes of anxiety were examined, social anxiety and worry, of which only the former has been previously examined as a criterion of ER strategies. Concerning affect, and despite possible objections to Cabello et al.'s proposition, it is also worthwhile to investigate whether the individualist vs. collectivist distinction can account for the inconsistent associations of ER strategies with affect. The study aimed to address this point by utilizing the multi-cultural composition of its sample.

## Method

### Participants and procedure

The sample consisted of 203 students and non-students (145 female), who were recruited via the subject pool of a large British university. Participants had a mean age of 22.7 years (*SD* = 5.7) and were primarily psychology and linguistics students, but students from other disciplines were also included. Non-students constituted 6.4% of the sample. All participants reported having obtained A level qualifications or an equivalent, while 20.7% had completed a bachelor's degree and 15.3% a Master's degree. The ethnic composition of the sample was 56.2% Caucasian, 22.7% Chinese, 6.4% Indian, and 14.7% had other or multi-ethnic backgrounds. English was reported as a native language by 65% of the participants, while 90.4% had been speaking English for more than 5 years.

Data collection occurred anonymously via an online survey system. Therefore, missing responses could be highlighted to the participants for each of the measures. Participants received course credit for their time and were included in a draw for gift cards. The investigation had obtained ethical approval and participants gave their informed consent prior to beginning the study.

### Measures

Internal consistency scores derived from the study sample appear in Table 1. All variables reached acceptable to high alphas, ranging from 0.69 to 0.94.

**Table 1:** **Correlations and coefficient alphas for ERQ subscales, the big five, and criterion variables**.

	**1**	**2**	**3**	**4**	**5**	**6**	**7**	**8**	**9**	**10**	**11**	**12**	**13**	**14**	**15**	**16**
1. ERQ CR	(0.85)															
2. ERQ ES	0.18^**^	(0.79)														
3. Neuroticism	−0.37^***^	−0.14^*^	(0.84)													
4. Extraversion	0.05	−0.33^***^	−0.18^**^	(0.84)												
5. Openness	0.11	0.02	0.10	0.19^**^	(0.83)											
6. Agreeableness	0.24^***^	−0.21^**^	−0.31^***^	0.18^*^	0.16^*^	(0.69)										
7. Conscientiousness	0.15^*^	−0.08	−0.18^**^	0.15^*^	0.07	0.23^***^	(0.78)									
8. Positive affect	0.23^***^	−0.17^*^	−0.19^**^	0.43^***^	0.29^***^	0.25^***^	0.26^***^	(0.89)								
9. Negative affect	−0.23^***^	0.09	0.56^***^	−0.08	0.12	−0.34^***^	−0.25^***^	0.13	(0.91)							
10. Worry	−0.30^***^	−0.14^*^	0.79^***^	−0.16^*^	0.07	−0.16^*^	−0.15^*^	−0.09	0.47^***^	(0.94)						
11. Social anxiety	−0.09	0.17^*^	0.49^***^	−0.59^***^	0.00	−0.11	−0.07	−0.28^***^	0.28^***^	0.42^***^	(0.78)					
12. Rumination	−0.08	0.03	0.57^***^	−0.15*	0.25^***^	−0.03	−0.23^***^	−0.15*	0.32^***^	0.56^***^	0.39^***^	(0.91)				
13. Reflection	0.008	0.05	0.13	0.01	0.62^***^	0.11	0.02	0.12	0.21^**^	0.05	0.07	0.29^***^	(0.90)			
14. Flexibility	0.35^***^	−0.08	−0.65^***^	0.22^**^	0.00	0.30^***^	0.36^***^	0.19^**^	−0.65^***^	−0.56^***^	−0.33^***^	−0.51^***^	−0.15^*^	(0.88)		
15. PNE	0.22^***^	−0.04	−0.23^***^	0.26^***^	0.17^*^	0.15^*^	0.25^***^	0.41^***^	−0.05	−0.17^*^	−0.24^***^	−0.15^*^	0.10	0.19^**^	(0.72)	
16. CSA	0.29^***^	0.06	−0.11	0.06	0.23^***^	0.27^***^	0.28^***^	0.18^*^	−0.25^***^	−0.01	0.07	0.08	0.13	0.28^***^	0.05	(0.85)

*N = 200–203. Coefficient alphas are presented in parentheses along the diagonal. ERQ, Emotion Regulation Questionnaire (Gross and John, 2003); CR, cognitive reappraisal; ES, expressive suppression; PNE, preventing negative emotions; CSA, constructive self-assertion*.

* *p <0.05*.

** *p <0.01*.

*** *p <0.001*.

#### Predictors

##### ER

The 10-item ERQ (Gross and John, 2003) was used to examine the use of two regulatory strategies: CR and ES. Respondents indicate their tendency toward reappraisal (six items) and suppression (four items) through a seven-point Likert scale, ranging from one (*strongly disagree*) to seven (*strongly agree*). Independent scores are computed for each ER strategy.

##### Big Five

The Big Five personality factors were evaluated using the Big Five Inventory (John et al., 1991). The factors of Neuroticism (eight items), Extraversion (eight items), Openness (10 items), Agreeableness (nine items), and Conscientiousness (nine items) are examined through 44 Likert-type items, ranging from one (*disagree strongly*) to five (*agree strongly*).

#### Emotion-laden criteria

##### Positive and Negative Affect

The Positive and Negative Affect Schedule (Watson et al., 1988) was used to measure positive affect (10 items) and negative affect (10 items). Respondents rate the frequency of 20 items describing their mood via a five-point scale, worded “*Very slightly or not at all*” (1) to “*Extremely*” (5).

##### Worry

The Penn State Worry Questionnaire (Meyer et al., 1990) provided an assessment of the trait of worry as an independent component of generalized anxiety. The PSWQ consists of 16 Likert-type items, ranging from one (*Not at all typical of me*) to five (*Very typical of me*), and investigates worry as a single-factor construct.

##### Social Anxiety

The Social Anxiety subscale from the Self-Consciousness Scale (Fenigstein et al., 1975) was utilized to assess generalized discomfort in social contexts. The subscale is composed of six items, and responses are recorded via a five-point scale ranging from zero (*Extremely uncharacteristic*) to four (*Extremely characteristic*).

#### Cognitive criteria

##### Rumination and reflection

The tendency of individuals to ruminate about and reflect on their self was measured using the Rumination–Reflection Questionnaire (Trapnell and Campbell, 1999). Rumination involves becoming preoccupied with perceived threats and anxieties, while reflection relates to introspection motivated by epistemic curiosity. The RRQ contains 24 items in total and is composed of two subscales: rumination (12 items) and reflection (12 items). Items are scored via a five-point Likert scale, worded “*Strongly disagree*” (1) to “*Strongly agree*” (5). An independent mean score is computed for each subscale.

##### Experiential flexibility

Experiential flexibility was measured using the revised Acceptance and Action Questionnaire (Bond et al., 2011), examining avoidance (vs. acceptance) of ongoing experiences as a means of behavioral regulation. The scale includes 10 items in total, with individuals rating their tendency toward experiential acceptance through a seven-point scale ranging from one (*Never true*) to seven (*Always true*).

##### Mindful Coping

The coping facets of mindfulness were examined using two subscales of the Mindful Coping Scale (Tharaldsen and Bru, 2011), namely “preventing negative emotions” and “constructive self-assertion.” The first subscale assesses individual ability in generating positive and limiting negative emotions, and is examined by 5 Likert-type items, ranging from one (never/hardly ever) to five (always). The second subscale, also examined by five items, reflects the extent to which an individual sufficiently supports his interests during interpersonal communication, while tending to the nature of the relationship.

## Results

### Data screening and preliminary analyses

Three participants did not complete all of the measures. Since pairwise deletion was used to calculate bivariate correlations, correlations coefficients are based on 200–203 participants, whereas all regression results are based on the data from 200 participants. Table 1 includes ranges of missing data points (proportions) for the items corresponding to each variable. As participants were notified of missing responses after completing each measure, the proportions of missing data were small, with no missing data points on any of the ERQ items. The maximum percentages of missing item scores for the other measures were 2.5% for the Big Five Inventory, 1.5% for emotion-laden criteria, and 1.0% for cognitive criteria. Scale scores were therefore derived from the average of completed items.

Proportions of male and female participants did not differ significantly between British (32.8% male) and Chinese participants (23.9% male), χ^2^(1, *N* = 110) = 1.03, *p* = 0.31. Further, the mean ages of British (*M* = 23.2, *SD* = 8.0) and Chinese participants (*M* = 21.3, *SD* = 1.7) were similar and not significantly different, *t*_(70.77)_ = 1.88, *p* = 0.06, adjusting for unequal variances. Thus, neither gender nor age were controlled in the main analyses.

### Bivariate correlations

The bivariate correlation coefficients for the ERQ, the BFI, and emotion-laden and cognitive criteria are shown in Table 1. The two ERQ subscales were largely independent, showing a weak positive correlation. Concerning higher-order personality factors, CR had a moderate negative correlation with Neuroticism and modest correlations with Conscientiousness and Agreeableness. ES had a moderate negative association with Extraversion and weak negative correlations with Agreeableness and Neuroticism.

Positive affect correlated positively with CR and negatively with ES, while negative affect correlated negatively with CR. All associations were modest in size. Notably, the association between negative affect and ES was non-significant. Worry showed a moderate negative correlation with CR, while social anxiety was not significantly related to CR. ES was weakly associated with social anxiety and showed a weak negative association with worry.

Following Cabello et al.'s (2012) suggestion that the observed variability in the relationship of CR and ES with affect could be based on cultural features of collectivist countries, correlations between the ERQ subscales and affect for two major components of the sample, namely British (31.5%) and Chinese (22.7%) participants, were examined. The correlations are displayed in Table 2. According to the classification of individualism vs. collectivism by Hofstede et al. (2010), the UK is a highly individualist country, scoring 89 out of 100 on the individualism index. In contrast, China as a strongly collectivist country has a score of 20 out of 100 on this index. The results for the UK showed CR to be related to reduced negative affect (*r* = −0.27, *p* = 0.03), but not increased positive affect (*r* = 0.24, *p* = 0.06), while ES was related to reduced positive affect (*r* = −0.33, *p* = 0.008), but not increased negative affect (*r* = −0.08, *p* = 0.55). For China, CR was associated with reduced negative affect (*r* = −0.34, *p* = 0.02), but not increased positive affect (*r* = 0.13, *p* = 0.41), and ES was not significantly associated with either positive (*r* = −0.01, *p* = 0.94) or negative affect (*r* = 0.09, *p* = 0.57).

**Table 2:** **Correlations between ERQ subscales and affect across samples from different backgrounds**.

	**Cognitive reappraisal**				**Expressive suppression**			
	**US**	**UK**	**China**	**Brazil**	**US**	**UK**	**China**	**Brazil**
Positive affect	0.42^*^	0.24	0.13	0.37^*^	−0.33^*^	−0.33^*^	−0.01	0.04
Negative affect	−0.51^*^	−0.27^*^	−0.34^*^	−0.34^*^	0.39^*^	−0.08	0.09	−0.12

*US sample results stem from Gross and John (2003). Results for the UK and China are based on the cultural subgroups of the sample of the present study. Brazilian sample results stem from Batistoni et al. (2013) ERQ, Emotion Regulation Questionnaire (Gross and John, 2003)*.

* *p <0.05*.

With regards to cognitive criteria, CR demonstrated a moderate positive association with experiential flexibility and modest positive associations with preventing negative emotions and constructive self-assertion. ES did not correlate with any of these criteria.

### Hierarchical regression analyses

In order to examine the incremental validity of the ERQ subscales over the Big Five personality factors, a 2-step hierarchical regression analysis was performed for each criterion. The criteria were first regressed on the Big Five (Step 1) and subsequently on CR and ES (Step 2). The results for emotion-laden and cognitive criteria can be found in Tables 3, 4, respectively. Because the compared variables (Big Five, ERQ) differed in number of predictors, and, thus, in degrees of freedom, the adjusted *R*^2^-values are used here to estimate the effect sizes of the ERQ as a whole. The beta weights shown in the tables are standardized.

**Table 3:** **Hierarchical regression analyses predicting emotion-laden criteria from the Big Five (Step 1) and the two ERQ subscales (Step 2)**.

	**Positive affect**		**Negative affect**		**Worry**		**Social anxiety**	
Step 1: Big Five	*F*_(5, 194)_ = 15.37^***^		*F*_(5, 194)_ = 22.77^***^		*F*_(5, 194)_ = 65.79^***^		*F*_(5, 194)_ = 41.89^***^	
Step 2: ERQ subscales	*F*_(7, 192)_ = 11.89^***^		*F*_(7, 192)_ = 17.32^***^		*F*_(7, 192)_ = 46.58^***^		*F*_(7, 192)_ = 30.47^***^	
Predictor	β	*R*^2^_*Adj*_ (Δ*R*^2^)	β	*R*^2^_*Adj*_ (Δ*R*^2^)	β	*R*^2^_*Adj*_ (Δ*R*^2^)	β	*R*^2^_*Adj*_ (Δ*R*^2^)
(Step 1)		0.27		0.35		0.62		0.51
N	−0.10		0.47^***^		0.82^***^		0.43^***^	
E	0.33^***^		0.04		−0.02		−0.55^***^	
O	0.21^**^		0.11		−0.03		0.04	
A	0.09		−0.19^**^		0.11^*^		0.10	
C	0.16^*^		−0.14^*^		−0.02		0.07	
(Step 2)		0.28 (0.01)		0.36 (0.01)		0.62 (0.00)		0.51 (0.00)
CR	0.15^*^		−0.04		−0.02		0.05	
ES	−0.06		0.15^*^		0.00		0.07	

*N = 200. All beta weights are standardized. ERQ, Emotion Regulation Questionnaire (Gross and John, 2003); N, Neuroticism; E, Extraversion; O, Openness; A, Agreeableness; C, Conscientiousness; CR, cognitive reappraisal; ES, expressive suppression*.

* p <0.05;

** p <0.01;

*** *p <0.001*.

**Table 4:** **Hierarchical Regression Analyses Predicting Cognition and Mindful Coping Criteria from the Big Five (Step 1) and the two ERQ Subscales (Step 2)**.

	**Rumination**		**Reflection**		**Experiential flexibility**		**Preventing negative emotions**		**Constructive self-assertion**	
Step 1: Big Five	*F*_(5, 194)_ = 27.31^***^		*F*_(5, 194)_ = 26.29^***^		*F*_(5, 194)_ = 37.43^***^		*F*_(5, 194)_ = 7.33^***^		*F*_(5, 194)_ = 7.32^***^	
Step 2: ERQ subscales	*F*_(7, 192)_ = 21.09^***^		*F*_(7, 192)_ = 18.66^***^		*F*_(7, 192)_ = 30.36^***^		*F*_(7, 192)_ = 5.75^***^		*F*_(7, 192)_ = 6.88^***^	
Predictor	β	*R*^2^_*Adj*_ (Δ*R*^2^)	β	*R*^2^_*Adj*_ (Δ*R*^2^)	β	*R*^2^_*Adj*_ (Δ*R*^2^)	β	*R*^2^_*Adj*_ (Δ*R*^2^)	β	*R*^2^_*Adj*_ (Δ*R*^2^)
(Step 1)		0.40		0.39		0.48		0.14		0.13
N	0.56^***^		0.06		−0.59^***^		−0.18^*^		−0.04	
E	−0.09		−0.11		0.06		0.17^*^		−0.06	
O	0.20^**^		0.63^***^		0.02		0.15^*^		0.20^**^	
A	0.17^**^		0.05		0.05		0.00		0.18^*^	
C	−0.17^**^		−0.01		0.23^**^		0.19^**^		0.22^**^	
(Step 2)		0.41 (0.01^*^)		0.38 (−0.01)		0.51(0.03^**^)		0.14 (0.00)		0.17 (0.04^**^)
CR	0.10		0.03		0.14^*^		0.13		0.20^**^	
ES	0.12		0.00		−0.18^**^		−0.01		0.08	

*N = 200. All beta weights are standardized. ERQ, Emotion Regulation Questionnaire (Gross and John, 2003); N, Neuroticism; E, Extraversion; O, Openness; A, Agreeableness; C, Conscientiousness; CR, cognitive reappraisal; ES, expressive suppression*.

* p <0.05;

** p <0.01;

*** *p <0.001*.

Step 1 of the analyses showed the Big Five to account for significant variance in all emotion-laden criteria, ranging from 27% (positive affect) to 62% (worry). Positive affect was significantly predicted by high Extraversion, Openness, and Conscientiousness, while high Neuroticism and low Agreeableness and Conscientiousness were predictors of negative affect. Neuroticism and Agreeableness showed significant positive beta weights for worry, and social anxiety was significantly predicted by low Extraversion and high Neuroticism.

The addition of the two ERQ subscales in the regression model did not significantly increase the overall variance explained for any of the emotion-laden criteria (Table 3), despite significant beta coefficients for some. CR had a positive and significant beta in predicting positive affect (β = 0.15), while ES had a significant positive beta in the prediction of negative affect (β = 0.15). No significant betas were noted for worry and social anxiety.

As shown in Table 4, the Big Five also accounted for considerable variance of the cognitive criteria (*R*^2^ = 13–48%). Increased Neuroticism, Openness, Agreeableness, and reduced Conscientiousness were significant predictors of rumination. In contrast, only increased Openness predicted reflection scores. Experiential flexibility was predicted negatively by Neuroticism and positively by Conscientiousness. Finally, increased Openness, Extraversion and Conscientiousness, and reduced Neuroticism predicted preventing negative emotions, while Openness, Conscientiousness, and Agreeableness positively predicted constructive self-assertion.

The ERQ subscales explained unique variance above and beyond the Big Five in three of the cognitive criteria (Table 4): rumination (Δ*R*^2^ = 1%), experiential flexibility (Δ*R*^2^ = 3%), and constructive self-assertion (Δ*R*^2^ = 4%). Despite the overall significant effects for rumination, CR and ES both had non-significant beta-weights. Moreover, CR accounted for significant incremental variance in experiential flexibility (β = 0.14) and constructive self-assertion (β = 0.20) and approached significance in predicting preventing negative emotions (β = 0.13, *p* = 0.07). ES had a significant predictive effect on experiential flexibility (β = −0.18).

Following the significant contribution of the ERQ subscales for experiential flexibility and constructive self-assertion, a *post-hoc* analysis was conducted to examine if the ERQ maintains its incremental effects beyond both the Big Five and general emotional states (positive and negative affect) for those two criteria. In this analysis, the Big Five were entered at Step 1, positive and negative affect at Step 2, and the two ERQ subscales at Step 3. The results showed that the ERQ scores still explained incremental variance in experiential flexibility (Δ*R*^2^ = 1.5%) and constructive self-assertion (Δ*R*^2^ = 4%) with both the Big Five and positive and negative affect in the equation. In terms of beta weights, a similar pattern as in the initial regression model was observed; both CR (β = 0.11) and ES (β = −0.11) made significant contributions to experiential flexibility, while only CR (β = 0.16) significantly contributed to constructive self-assertion. While there was no substantial change in these values between the two models, the variance explained by the ERQ for experiential flexibility was halved when affect was included as a predictor (1.5% change).

## Discussion

The present study is among the first to examine the incremental validity of ER strategies measured by the ERQ. Previous research (Páez et al., 2013) showed incremental effects for ES, but not CR, in predicting criterion variance over the Big Five personality factors in happiness and psychological well-being. Moreover, this study also sought to extend, and partially replicate the evidence for the measure's criterion validity.

### Criterion validity

As expected, CR was significantly related to high positive and low negative affect, and ES demonstrated a negative association with positive affect. Nevertheless, ES was not associated with increased negative affect, an observation that joins the growing literature (Kashdan et al., 2006; Balzarotti et al., 2010; Cabello et al., 2012; Batistoni et al., 2013) and contradicts the initial findings by Gross and John (2003). The observed non-significant association between ES and negative affect is perhaps the least surprising result, given the variability in past findings. Despite contradicting the results from Gross and John (2003), this finding is in line with experimental investigations suggesting that ES has no effect on negative affect (e.g., Gross and Levenson, 1997; Gross, 1998). In fact, Gross and John's (2003) initial hypothesis was that their investigation would reveal a non-significant relationship.

Correlations between affect and the ERQ subscales for specific cultural subgroups of the sample (British and Chinese participants) did not support Cabello et al.'s (2012) proposition that variation in the relationship between the ERQ subscales and affect can be attributed to the level of individualism vs. collectivism of the study samples. According to Hofstede et al. (2010), the UK is a heavily individualistic country, while China is strongly collectivistic. Table 2 includes the results of samples from the US (Gross and John, 2003) and Brazil (Batistoni et al., 2013), two countries falling on differing ends of the individualist vs. collectivist continuum (Hofstede et al., 2010). The UK subgroup demonstrated non-significant associations for CR and positive affect and for ES and negative affect, contradicting the US-based findings (Gross and John, 2003). Likewise, the results from Chinese participants did not replicate the positive association between CR and positive affect observed in the Brazilian sample (Batistoni et al., 2013) and, although non-significant, the correlation coefficients of ES and negative affect were in opposite directions for China and Brazil.

With regard to the remaining emotion-laden criteria, social anxiety was positively related to ES and unrelated to CR, while worry was negatively related to CR, supporting previous findings (Kashdan et al., 2006; Wiltink et al., 2011). A surprising result was the significant association of use of ES with reduced worry, which contradicts foregoing findings that support the relation of ES with various types of anxiety (Kashdan et al., 2006; Wiltink et al., 2011).

The majority of the findings for the cognitive criteria were not in consistent with expectations. Contrary to Gross and John (2003), neither ERQ strategy significantly predicted rumination, though both relationships were in the anticipated direction. Moreover, CR was positively associated with experiential flexibility but not with reflection, while none of the associations of ES with cognitive criteria were significant, contradicting the hypotheses. Nevertheless, predictions of a positive association of CR with preventing negative emotions and constructive self-assertion were supported.

Although the prediction was that CR would be unrelated to experiential flexibility, the observed positive association between the two variables is certainly interpretable. Avoidance of present experience is a process characterized by an attempt to distance oneself from particular experiences and their emotional, cognitive and physiological components (Hayes et al., 2004). Reinterpreting one's cognitions regarding an experience instead of evading it could therefore, dissolve the rigidity generated by avoidance and enhance flexibility. Moreover, the successful management of emotions, and decreasing emotional reactivity in particular, is a key part of the traditional Buddhist concept of mindfulness (Kumar, 2002) and an important component of mindfulness-based interventions (e.g., Linehan, 1993).

Some of the unanticipated relationships recorded here might have resulted from characteristics relating to the form and function of the particular criterion at hand. For example, worry diverges from other anxiety subtypes, involving a preparatory problem-solving mechanism that is associated with fear processing and that has decisive effects on the reaction of the individual to a worry-evoking situation (Borkovec et al., 1983). Suppressing the expression of worry could, thus, be beneficial in the long run, by preventing it from influencing one's actions. Similarly, rumination might entail both profitless and constructive cognitive mechanisms (Treynor et al., 2003). The lack of association of both strategies with rumination could be attributed to the multifaceted nature of rumination. Hence, it seems worthwhile to examine the relationship of the ERQ subscales with specific rumination subtypes, before well-founded hypotheses regarding the association of adaptive and maladaptive ER strategies with the global rumination construct can be formulated.

### Incremental validity

Relationships between the ERQ subscales and the Big Five were partially consistent with those reported by Gross and John (2003). CR correlated positively with Agreeableness and Conscientiousness, and showed a moderate negative correlation with Neuroticism. For ES, the expected moderate negative correlation with Extraversion was observed, along with a negative correlation with Agreeableness. Nevertheless, there were also numerous divergent results. The relationships of CR with Extraversion and Openness were non-significant, while ES showed no association with Conscientiousness or Openness.

The observed negative association of ES with Neuroticism is in line with some previous studies (Balzarotti et al., 2010; Cabello et al., 2012), but also deviates from previously observed non-significant (Gross and John, 2003; Páez et al., 2013) or positive associations (Gresham and Gullone, 2012) between the two variables. Interestingly, although there have been theoretical accounts of both positive and non-significant associations (John and Gross, 2004; Gresham and Gullone, 2012), no explanation for a negative association has been proposed. A possible interpretation is that neurotic individuals have a reduced propensity for ER, which would explain the negative association of Neuroticism with both ERQ subscales, and their difficulty in dealing with emotional arousal (Costa and Widiger, 2002). This suggestion is also in agreement with the literature on emotional intelligence, which comprises emotional self-control and well-being at its core (Siegling et al., 2014b) and consistently shows a strong negative correlation with Neuroticism (e.g., Siegling et al., 2014a).

The results provided some support for the incremental validity of the ERQ of emotion-laden criteria vis-à-vis the Big Five personality factors. CR explained unique variance in positive affect and ES in negative affect. No incremental effects were observed for CR in predicting negative affect or for ES in predicting positive affect. In addition, none of the ERQ subscales explained unique variance in worry and social anxiety.

In terms of cognitive criteria, no significant contributions were observed for either of the ERQ subscales for rumination and reflection, although the two strategies showed a small but significant overall increase in variance explained in rumination. Nonetheless, ES negatively predicted experiential flexibility over the Big Five. Moreover, whereas Páez et al. (2013) only found ES to explain unique variance in construct-relevant criteria, CR also demonstrated incremental effects for experiential flexibility and constructive self-assertion, and approached significance in predicting preventing negative emotions. *Post-hoc* analyses of these results showed that the incremental contributions of CR and ES remained after including positive and negative affect in the regression model.

### Implications, limitations, and future directions

The study findings give some reason for optimism concerning the validity and utility of ER strategies and, to a lesser extent, the ERQ. However, although some of the findings of previous research were replicated, numerous associations of the ERQ subscales with the examined criteria either contradicted earlier findings, or did not correspond to expectations. These results suggest that further psychometric work on the ERQ is necessary before drawing conclusions about the validity of ER strategies. Of note, while CR significantly predicted nine out of the 14 variables in the study and also yielded a meaningful non-significant association with social anxiety, ES predicted only six, while two associations were in the opposite direction of that expected. Consequently, future studies should place particular importance on examining and providing evidence for the validity of this ERQ subscale.

It is important to keep in mind that a brief measure was used to assess the two broad ER strategies of CR and ES. Even though both subscales reached acceptable levels of internal reliability, they may not represent the respective ER strategies comprehensively. This limited coverage likely places an upper limit on the measure's associations with construct-relevant criteria. Consequently, stronger criterion and incremental validity results can be expected when employing more comprehensive measures for ERQ strategies. The ES subscale, which is based on only four items, was less likely than CR to live up to expectations, which supports the notion that the limited construct coverage of the ERQ impacted on the observed pattern of results. In any case, evidence on the criterion and incremental validity of ER strategies is still scarce and additional research is needed, presumably using more advanced measures than the ERQ in its current form.

Future research should seek to understand the factors that are responsible for the variability observed in some of the relationships of the ERQ with other variables. Aside from the importance of culture, which was addressed to some extent in the present study, attention should perhaps be given to the effects of age. Age is likely to affect ER processes, especially in early ages where successful ER is viewed as a critical developmental task (Cicchetti et al., 1991). The only investigation noting a non-significant association between ES and positive affect (Batistoni et al., 2013) involved a senior sample with a mean age of 67 years. Secondly, the one study reporting a positive association between Neuroticism and ES was that by Gresham and Gullone (2012), which relied on a sample comprised of children and adolescents ranging from 10 to 18 years of age.

Due to self-report measures being a discernible limitation when examining complex, ongoing processes, such as ER, it is of importance to investigate the validity of the ERQ in predicting more objectively-assessed criteria. Hence, the use of methods involving observation-based ratings from independent trained assessors is highly recommended. Another alternative to self-report measures in investigating the utility of ER strategies would be the use of “micro studies” (Hayes et al., 2006) examining and comparing the effectiveness of different psychotherapeutic interventions in countering negative emotional, cognitive, and behavioral states. Since ER strategies constitute essential components of the therapeutic process of various interventions (e.g., CR and cognitive behavioral therapy; Clark, 1999), therapy outcomes can provide an indication of their empirical and practical value.

### Conflict of interest statement

The authors declare that the research was conducted in the absence of any commercial or financial relationships that could be construed as a potential conflict of interest.
